# Case Report: Pathological confirmation and aggressive postoperative recurrence of WHO grade III rhabdoid meningioma

**DOI:** 10.3389/fmed.2025.1715197

**Published:** 2026-01-14

**Authors:** Rui Xu, Wei Wang, Zhen Wang, Kun Lian, Yongchao Gao, Pengyan Guo, Pengyu Qiao, Yongjun Gao

**Affiliations:** 1 Department of Neurosurgery, The Second Affiliated Hospital of Kunming Medical University, Kunming, China; 2 Department of Neurosurgery, Sixth People's Hospital of Chengdu, Chengdu, China

**Keywords:** case report, chemotherapy, postoperative recurrence, radiotherapy, rhabdoid meningioma

## Abstract

Rhabdoid meningioma (RM), a rare WHO grade III meningioma subtype, features high invasiveness, poor prognosis, and no effective therapies, often being misdiagnosed with other intracranial tumors clinically, such as brain metastases and WHO grade II meningiomas. We report a 39-year-old female admitted for 6-month headaches. Brain magnetic resonance imaging showed a well-circumscribed, heterogeneously enhancing tumor with severe peritumoral edema in the right temporal lobe, initially suspected to be a WHO grade II meningioma; RM was confirmed postoperatively. The tumor recurred 1 month after the first surgery, and the patient died of intracranial hypertension 1 month after the reoperation. This case highlights the need for immediate adjuvant chemoradiotherapy postoperatively.

## Introduction

RM represents a rare and aggressive subtype of meningioma, characterized by high invasiveness, rapid disease progression, and a strong tendency for early postoperative recurrence. First conceptualized by Perry et al. (1) and Kepes et al. (2) in 1998. RM was formally classified as a WHO grade III meningioma and recognized as a distinct subtype in the 2021 WHO (3, 4) Classification of Tumors of the Central Nervous System, underscoring its clinical and pathological significance. In contrast to conventional meningiomas, RM displays significant malignant potential and aggressiveness, which, combined with its rarity, makes it a diagnostic challenge—its radiological manifestations often overlap with those of other intracranial tumors, frequently leading to misdiagnosis in clinical practice. While over 200 cases of intracranial RM have been reported since 2000, recurrence, though common, typically follows a more indolent timeline; critically, no prior report has documented such an extremely rapid recurrence as observed in the present case, with tumor regrowth detected within 1 month of surgical resection.

## Case presentation

### History and examination

**Figure 1:**
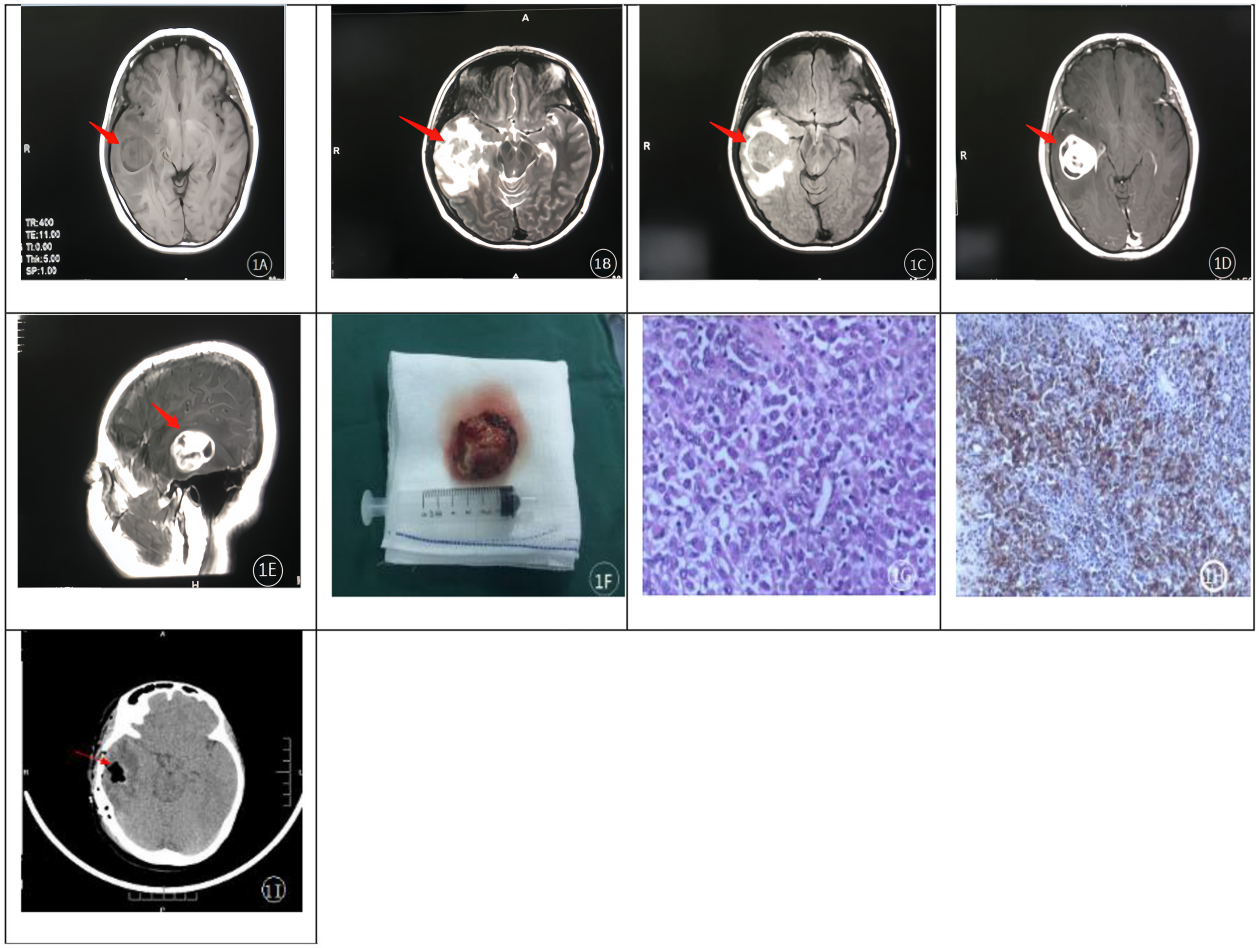
**(A–H)** shows cranial MRI axial images. T1-weighted **(A)** and T2-weighted **(B)** images revealed a mixed signal intensity in the right temporal region, with a slightly isointense signal on FLAIR imaging **(C)**. Extensive perilesional edema was observed on both T1- and T2-weighted sequences. Contrast-enhanced scans **(D, E)** demonstrated homogeneous enhancement of the lesion, with a small patchy cystic area within it. Pathological sections **(G, H)** showed eccentric, rhabdoid nuclei, prominent nuclear atypia, frequent mitotic figures, and multifocal necrosis. **(F)** shows an intraoperative photograph of the tumor resected during the initial surgery. 1 **(I)** shows the postoperative CT scan following tumor resection.

A 39-year-old female presented with intermittent headaches lasting over 6 months. She was alert (GCS 15/15) with intact orientation to person, place, and time, mild recent memory deficit, and an anxious mood. Cranial MRI revealed a large space-occupying lesion in the right temporal lobe, with a quasi-circular morphology measuring approximately 34 mm × 32 mm; imaging characteristics included mixed signal intensity on T1- and T2-weighted sequences (Figures 1A, B), isointense signal on fluid-attenuated inversion recovery (FLAIR) imaging (Figure 1C), and marked enhancement of the solid component on contrast-enhanced scans (Figures 1D, E). Significant perilesional edema was observed, accompanied by compression of the adjacent brainstem, lateral ventricle, and cerebral parenchyma, as well as a mild leftward midline shift, leading to a radiological differential diagnosis strongly favoring glioma. Neurological examination revealed the following findings: cranial nerves—right olfactory hypoesthesia, right visual acuity 20/40 (no homonymous hemianopsia), bilateral papilledema, bilateral 3 mm round pupils (light-reactive), right eye abduction limitation, right facial hypoesthesia, right sensorineural hearing mildly impaired, right hypoactive gag reflex plus mild dysphagia, right sternocleidomastoid/trapezius weakness, tongue deviation right; Motor—right upper/lower extremity hypertonia (Ashworth 1+) and mild weakness (5–/5), left extremities 5/5, right hemiparetic gait, right limb ataxia; Sensory—right trunk/limb hypoesthesia to superficial/deep/cortical sensations; Reflexes—right biceps/triceps/brachioradialis/patellar/Achilles reflexes hyperactive (+++), left normal (++), right Babinski sign positive; Meningeal signs—neck stiffness, Kernig's, and Brudzinski's signs positive. These findings correlate with the right temporal mass, perilesional edema, increased intracranial pressure, and mild midline shift on imaging.

Upon admission, mannitol 125 mL intravenous (IV) q12h was administered as dehydration therapy for symptomatic relief of the patient's headache, followed by right temporal lesion resection. Intraoperatively, the tumor was noted to have its base at the posterior horn of the right lateral ventricle, with a grayish to pinkish-gray appearance, soft consistency, clear demarcation from surrounding brain tissue, and rich vascular supply (Figure 1F). The lesion was completely resected (Figure 1I) and sent for pathological examination. Microscopic analysis ( × 400 magnification) revealed diffuse infiltration of tumor cells with abundant cytoplasm, eccentrically placed nuclei with rhabdoid morphology, prominent nuclear atypia, visible nucleoli, frequent mitotic figures, and multifocal necrosis—features consistent with a rhabdoid-type malignant tumor (Figure 1G). Special staining demonstrated abundant reticular fibers, and immunohistochemical analysis showed positive expression of EMA (Epithelial Membrane Antigen) (+) and vimentin, along with SSTR-2 (–), STAT-6 (cytoplasmic +), BRAFV600E (VE1, partial +), and INI-1 (+) (Figure 1H). These findings confirmed the diagnosis of RM, WHO grade III. No adjuvant radiotherapy or chemotherapy was administered postoperatively. The postoperative dehydration regimen consisted of mannitol 125 mL IV q8h initiated on postoperative day 3 (POD 3), adjusted to 125 mL IV q12h on POD 7, and discontinued on POD 12. Neurological examination at discharge (postoperatively): cranial nerves—right olfaction improved vs prior, right visual acuity enhanced, bilateral papilledema resolved, bilateral 3 mm round pupils (mildly reactive), right eye abduction mildly limited (markedly improved), right facial sensation improved, mild right sensorineural hearing loss; Motor—right upper/lower extremity tone normal, mild weakness (5-/5), left extremities 5/5, right hemiparetic gait (no significant improvement), no right limb ataxia; Sensory—right trunk/limb sensation improved (superficial/deep/cortical); Reflexes—right biceps/triceps/brachioradialis/patellar/Achilles reflexes normal, right Babinski sign negative; Meningeal signs—neck supple, Kernig's/Brudzinski's signs negative.

**Figure 2:**
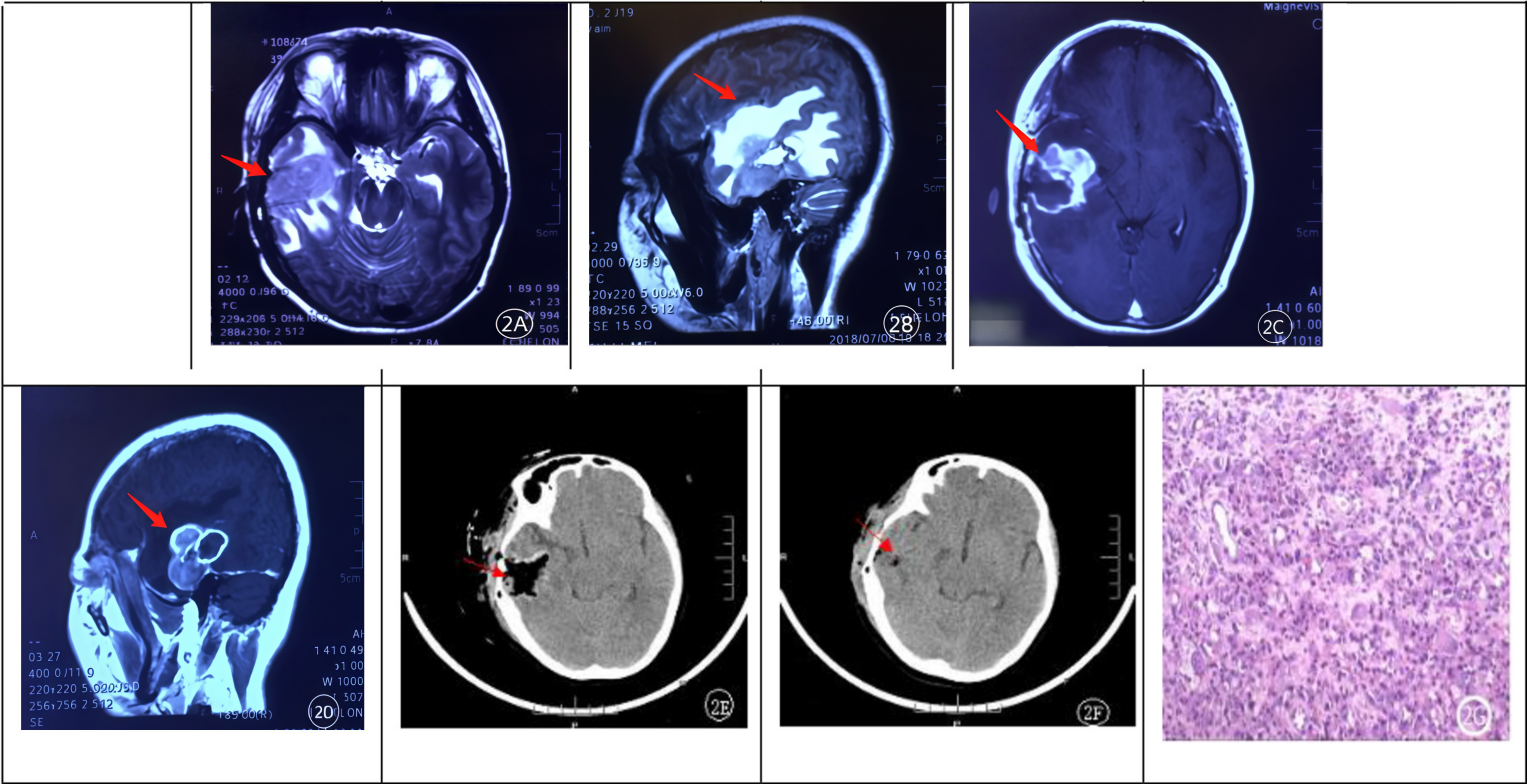
**(A–D)** illustrates tumor recurrence 1 month after the initial operation. MRI revealed extensive peritumoral edema and heterogeneous garland-like enhancement of the recurrent lesion after contrast administration. **(E, F)** demonstrated rapid tumor growth and cavity filling by the tumor at 6 hours and 1 week after re-resection, respectively. Postoperative pathological examination **(G)** confirmed recurrent rhabdoid meningioma.

One month after the initial resection, the patient developed sudden, intractable severe headaches at home and was emergently admitted. Except for the acutely exacerbated headaches, all other neurological symptoms were milder than those prior to the initial surgery. Emergency MRI confirmed tumor recurrence (Figures 2A–D). Upon emergency admission, dehydration therapy with mannitol 125 mL IV q8h was administered for two consecutive days, followed by urgent re-resection involving extended excision of the recurrent tumor and surrounding brain tissue. Six hours postoperatively, a repeat cranial CT scan demonstrated complete resection of the tumor (Figure 2E). Pathological examination following the second surgery further corroborated the pathological report from the first surgery (Figure 2G). Her symptoms improved transiently following the second surgery, with a neurological examination similar to that at discharge after the initial surgery; the postoperative dehydration regimen was consistent with that administered after the first operation. However, 1 week postoperatively, a repeat cranial CT scan revealed tumor recurrence (Figure 2F). Due to financial constraints, the patient and her family declined postoperative adjuvant radiotherapy and chemotherapy. Recurrent intracranial hypertension developed 1 month after the second surgery. Aggressive dehydration therapy was initiated following the onset of acute intracranial hypertension, consisting of 250 mL intravenous mannitol administered every 8 h, interspersed with 10 mg intravenous furosemide bolus between doses, and 4 mg intravenous dexamethasone given every 6 h for 7 days. In the absence of further surgical intervention, the patient succumbed to the disease a few days later.

## Discussion

### Clinical features

Meningioma is one common intracranial tumor with various morphologies and multiple subtypes. RM is listed as a rare subtype in the 2021 WHO classification of central nervous system tumors, accounting for less than 1% of all meningiomas due to its distinctive histopathological features and aggressive biological behavior (5). It belongs to WHO grade III meningioma and is classified as a malignant meningioma, alongside papillary and anaplastic meningiomas, owing to its high invasiveness, recurrence tendency, and poor prognosis (3, 4). RM originates from arachnoid epithelial cells, representing a malignant variant of meningioma characterized by rhabdomyocyte-like cellular morphology without evidence of striated muscle differentiation (6, 7). RM can be found at different age stages of life; however, literature reports indicate a higher prevalence in adolescents compared to the elderly, and a slight female predominance, which may be associated with the regulatory role of progesterone in the development and progression of meningiomas (8, 9). RM mainly arises in the parasagittal region, posterior fossa, cerebral convexity, and cerebellopontine angle, showing no significant difference from the common anatomical sites of intracranial meningiomas (10). In addition, some studies have noted that deletions of specific genetic regions, such as 22q11.2, correlate more closely with tumor behavior than histopathological findings alone (11). Among current research directions, gene-related studies may provide a promising approach to elucidate the underlying mechanisms of its abnormal clinical behaviors. In this case, the patient was a middle-aged female, with the lesion located in the right temporal lobe; headache and dizziness were the initial symptoms, rapid recurrence occurred 1 month after surgery, which is consistent with the clinical characteristics of RM reported in previous literature.

### Radiological features

Given the rarity of RM, large-scale radiological analyses based on prospective cohorts remain lacking in international literature, with current understanding primarily derived from retrospective case series and literature reviews. CT scans of RM show no pathognomonic features, limiting their utility in preoperative diagnosis. In contrast, MRI serves as the cornerstone of clinical differentiation, with several key albeit non-specific imaging hallmarks. RM typically exhibits isointense to slightly hypointense signals on T1-weighted imaging (T1WI) and heterogeneous signals on T2-weighted imaging (T2WI), with enhancement patterns (homogeneous or heterogeneous) on contrast-enhanced sequences directly correlating with the extent of intratumoral necrosis—a finding consistent with its high-grade malignant biology. A subset of cases presents with focal dural enhancement, termed the “dural tail sign,” which is hypothesized to result from tumor cell invasion into adjacent vasculature, inducing congestion and reactive hyperemia in the overlying dura mater. Regarding diffusion-weighted imaging (DWI), previous studies (11) have reported variable signal intensities (hyperintense or hypointense) in RM, which we propose may reflect a spectrum of malignancy-related cellularity: highly aggressive tumors with dense cellularity, reduced intercellular spacing, and marked nuclear atypia restrict water molecule diffusion, leading to DWI hyperintensity, while less malignant variants with lower cellularity and increased extracellular space exhibit hypointensity. Notably, unlike conventional meningiomas, RM lacks definitive MRI features that enable reliable distinction, emphasizing the need for integration with clinical context. A consistent radiological finding across reported cases, including ours, is prominent perilesional edema. In the present case, edema was particularly severe and adjacent to the brainstem—we speculate this may arise not only from mechanical compression of venous drainage (as previously proposed) but also from tumor-derived vascular permeability factors (e.g., vascular endothelial growth factor) (12, 13), which could exacerbate vasogenic edema in critical regions. RM is predominantly a solitary lesion, with radiological appearances categorized as cystic, solid-cystic, or purely solid. Intratumoral cystic changes are likely secondary to rapid cellular proliferation outpacing vascular supply, leading to intralesional necrosis, while peritumoral cystic changes may stem from cerebrospinal fluid redistribution caused by local hydrodynamic shifts due to mass effect (14, 15). Collectively, while no single MRI feature is diagnostic of RM, the combination of T2 heterogeneity, variable enhancement, prominent perilesional edema (especially when adjacent to critical structures), and cystic components may raise clinical suspicion, prompting earlier consideration of aggressive surgical planning and expedited pathological confirmation.

### Pathological features

Pathological Features: histologically, RM demonstrates three defining characteristics: (1) Morphological Heterogeneity with Clonal Evolution: Partial RM exhibits a biphasic cellular composition of typical meningothelial cells and rhabdoid cells, with focal transitions between these phenotypes. This histological continuum suggests clonal evolution driven by BAP1 gene inactivation (30–40% of cases) and SMARCB1 (INI1) loss (20–30% of cases), which disrupt chromatin remodeling and cell polarity (16). Recent studies highlight that BAP1 mutations correlate with aggressive behavior, as they impair histone H2AK119 deubiquitination, leading to aberrant gene silencing (17). (2) Invasive Growth Pattern and Angiogenic Mimicry: Rhabdoid cells display diffuse infiltration of brain parenchyma and frequently form papillary/vascular-centric structures. These pseudopapillary formations arise from tumor-derived vascular endothelial growth factor (VEGF) overexpression, which promotes endothelial fenestration and matrix metalloproteinase (MMP-9) activation (18). Notably, RM's angiogenic mimicry (vascular channel formation without endothelial lining) correlates with VEGFR-2 hyperactivation and AQP4 upregulation, exacerbating peritumoral edema in brainstem-adjacent tumors (19). (3) Cytological Malignancy and Necrotic Signature: Rhabdoid cells are characterized by large eosinophilic cytoplasm, pleomorphic nuclei with prominent nucleoli, and frequent intranuclear pseudoinclusions. Focal necrosis and ≥5 mitoses/10 HPF are diagnostic hallmarks, linked to TERT promoter mutations (5–10% of cases) and TP53 dysfunction (20). The 2024 WHO classification emphasizes that CDKN2A/B homozygous deletion (present in 15–20% of RM) confers worse prognosis, even in tumors lacking overt histological atypia (16).

### Immunophenotype

The immunophenotypic profile of RM tumor cells are characterized by the consistent co-expression of EMA and Vimentin, a feature that has been widely validated across international studies. Vimentin typically exhibits a diffuse cytoplasmic positivity, which is thought to be associated with the maintenance of cytoskeletal integrity during mesenchymal differentiation (21, 22); in contrast, EMA shows partial membranous staining, a pattern that may be linked to heterogeneous epithelial-mesenchymal transition (EMT) dynamics—a process previously implicated in tumor invasiveness. Notably, the expression patterns of these two markers in the present patient are consistent with such established findings. Concurrently, Desmin, GFAP, and SMA are consistently absent in RM tumor cells, further supporting the diagnostic specificity. Although Desmin is recognized as a sensitive marker for muscle differentiation, its clinical utility is limited by poor specificity, as cross-reactivity has been reported in other sarcomas such as leiomyosarcoma. In contrast, Myogenin and MyoD1 serve as more definitive diagnostic markers for RM, given their role as key regulators of skeletal muscle lineage commitment (23); nuclear expression of these transcription factors is considered pathognomonic for RMS and facilitates differentiation from morphologically similar entities such as Ewing sarcoma and neuroblastoma. Additionally, CD99 (24, 25), a cell surface glycoprotein, exhibits strong expression in Ewing sarcoma but is typically absent or weakly expressed in RM. This differential expression profile, when integrated with EMA/Vimentin co-expression patterns, provides valuable diagnostic cues for distinguishing RM from other small round cell tumors in clinical practice.

### Differential diagnosis

Accurate differential diagnosis of RM relies on integrating radiological features, histopathological findings, and molecular markers. Key differential entities and their distinguishing characteristics are as follows: (1) Glioma: gliomas predominantly arise in deep brain structures (e.g., cerebral hemispheres, thalamus) and are typically characterized by heterogeneous signal intensity on MRI, accompanied by extensive peritumoral edema and significant mass effect. Contrast-enhanced imaging may show variable enhancement (from obvious to subtle), which can overlap with RM. However, gliomas exhibit distinct molecular signatures: high-grade gliomas (e.g., glioblastoma) often harbor IDH1/2 mutations, EGFR amplification, or MGMT promoter methylation, whereas RM lacks these alterations. Additionally, gliomas frequently show GFAP positivity in immunohistochemistry, which is typically absent or weak in RM, aiding differentiation (26); (2) Primary central nervous system lymphoma (PCNSL): PCNSL demonstrates characteristic imaging features: isointense or slightly hypointense signals on T1WI, isointense or slightly hyperintense signals on T2WI, hypointense or isointense signals on FLAIR, and hyperintense signals on DWI (attributed to high cellularity causing restricted diffusion). Contrast-enhanced scans usually show massive, nodular, or patchy homogeneous enhancement, with occasional heterogeneous or ring-enhancing patterns. Meningeal dissemination presents as diffuse meningeal enhancement. Immunophenotypically, PCNSL is almost always B-cell derived, with CD20 positivity, which is not expressed in RM. Moreover, PCNSL often exhibits MYD88 mutations, a marker absent in RM, further facilitating differentiation (27); (3) Metastatic tumors: metastatic tumors typically display long T1 and long T2 signals on MRI, with garland-like enhancement on contrast scans, mimicking RM. However, they are distinguished by their clinical context: most patients have a known primary malignancy (e.g., lung, breast, or colorectal cancer), and lesions are often multiple and peripherally located (consistent with hematogenous spread). Radiologically, metastatic tumors frequently show “small lesions with large edema” (a hallmark of metastatic disease), whereas RM tends to present as a single mass with less extensive edema. Molecularly, metastatic lesions retain genetic alterations of the primary tumor (e.g., KRAS mutations in colorectal metastases), which are absent in RM (28); (4) A typical teratoid/rhabdoid tumor (AT/RT): both RM and AT/RT contain rhabdoid tumor cells and may show positivity for EMA, Vimentin, and occasionally Desmin or GFAP, leading to diagnostic overlap. However, AT/RT has distinct clinical and molecular features: it primarily affects infants and young children (< 3 years), with a predilection for the posterior fossa and supratentorial regions. Genetically, AT/RT is pathognomonic for inactivation of the SMARCB1 (INI1) gene (via 22q deletion or mutations) (29), resulting in loss of INI1 protein expression—an alteration rarely observed in RM. Recent studies have further identified SMARCA4 mutations as a secondary driver in some AT/RT cases, which are not associated with RM. Thus, INI1 immunohistochemistry (negative in AT/RT, positive in RM) remains the gold standard for differentiation (30).

### Treatment and prognosis

RM, a WHO grade III malignancy, is characterized by aggressive local invasion, rapid recurrence, and dismal outcomes, with a median survival of often < 12 months even following aggressive therapy. Despite its clinical urgency, standardized treatment guidelines remain lacking due to its extreme rarity (accounting for < 1% of all meningiomas) and limited research data. Current management strategies for rhabdoid meningiomas (RM) are thus extrapolated from high-grade meningioma protocols and informed by emerging molecular insights: surgical resection remains the cornerstone of curative intent, with gross total resection (GTR) associated with improved progression-free survival. Notably, the temporal lobe localization of our case afforded the potential for radical resection (e.g., extended infiltration zone excision or temporal lobectomy), though this approach demanded a careful balance between radicality and neurological safety—specifically mitigating risks of aphasia or hemiparesis. Supported by relevant RM-specific surgical literature, our clinical decision prioritized maximal safe resection, aiming to maximize tumor clearance while preserving critical neurological function (17). However, in contrast to the anatomical accessibility observed in our temporal lobe case, RM's inherent infiltrative growth pattern and frequent involvement of critical neuroanatomical structures (e.g., skull base, cavernous sinus) often preclude definitive radical excision in most cases, thereby necessitating radiotherapy and neoadjuvant chemotherapy (NACT) to downsize tumors and enhance resectability.

Postoperative radiotherapy remains a cornerstone of local disease control for meningiomas, as chemotherapy has not been proven to confer convincing evidence-based therapeutic efficacy for this disease—aligning with the clinical consensus that surgery combined with radiotherapy constitutes the first-line treatment strategy. Compared to conventional fractionated radiation, modern techniques including intensity-modulated radiation therapy (IMRT) and proton therapy offer superior precision in targeting residual tumor tissue while minimizing irradiation of adjacent normal structures (e.g., cranial nerves, brain parenchyma), translating to reduced treatment-related toxicity and improved tolerability, particularly for incompletely resected or high-risk cases.

Chemotherapy regimens, including the VAC (vincristine, dactinomycin, cyclophosphamide) or IE (ifosfamide, etoposide) protocols used in pediatric rhabdoid tumors, are commonly employed, though response rates remain low (20–30%) (17, 31); recent studies highlight the potential of dose-dense chemotherapy in high-risk patients, albeit with its applicability limited by myelosuppression and organ toxicity. Notably, NACT retains clinical relevance beyond objective response rates: it facilitates tumor downstaging to improve R0 resection rates (32, 33), suppresses micrometastases to prolong progression-free survival (PFS) (34, 35), and is guided by multidisciplinary individualized benefit-risk stratification (36). Modern supportive care mitigates quality of life (QoL) impairment, with most patients maintaining baseline QoL during treatment (37), and responders often experience post-treatment QoL improvement (35).

The low response rate underscores the need for optimized chemotherapy strategies, including second-line options for recurrent, refractory, or unresectable RM: temozolomide (TMZ), a widely used alkylating agent, serves as a key second-line therapy, supported by small-sample studies and case reports despite limited large-scale prospective evidence (38). While TMZ monotherapy yields modest overall response rates (median PFS ~5 months in refractory cases), preclinical data suggest synergistic antitumor effects when combined with PARP inhibitors (e.g., talazoparib) (39), and the MGMT gene promoter methylation status may act as a predictive biomarker for efficacy.

Beyond traditional cytotoxic therapies, molecular profiling is driving novel therapeutic approaches for sporadic RM. Targeted kinase inhibition for sporadic RM: a pediatric sporadic RM case harboring a BRAF-V600E mutation achieved durable remission with the combination of dabrafenib (a BRAF inhibitor) and trametinib (a MEK inhibitor). This genotype-driven regimen is currently under evaluation in early-phase trials specifically for BRAF-mutant sporadic meningiomas, providing a potential precision therapy option for this subtype (40). In contrast to sporadic RM, targeted therapy research for inherited neurofibromatosis type 2 (NF2)-related meningiomas (a distinct entity associated with germline NF2 gene mutations, not sporadic pathogenesis) has yielded separate findings: the dual mTORC1/2 inhibitor vistusertib demonstrated disease stabilization in 94% of NF2-related meningiomas in a prospective phase II trial. However, this agent has not been validated for sporadic RM—its dose-limiting toxicities (e.g., stomatitis, fatigue) require optimization even for NF2-related cases (41). For sporadic RM, preclinical studies further support the potential of PI3K/MEK co-targeting (e.g., alpelisib plus trametinib), which synergistically inhibits proliferation in RM cell lines. This strategy is independent of NF2-related pathways and remains a key research direction for sporadic high-grade meningiomas.

For immunotherapy, a phase II trial (NCT02648997) is assessing nivolumab with or without ipilimumab in recurrent meningiomas, with preliminary data showing durable responses in mismatch repair-deficient tumors; although RM-specific results are pending, PD-L1 expression in approximately 30% of high-grade meningiomas suggests potential efficacy, and preclinical models of glioma and melanoma brain metastases have demonstrated enhanced T-cell infiltration and improved survival when propranolol (a β-adrenergic blocker) is combined with checkpoint inhibitors—a strategy now being translated to meningioma trials. Additionally, among epigenetic modulators, sequential inhibition of MEK (using trametinib) followed by MCL-1 (using s63845) has been shown to overcome treatment resistance by blocking adaptive anti-apoptotic pathways in RM cells (42).

## Conclusion

RM, a rare WHO grade III meningioma, exhibits extremely aggressive biological behavior—evidenced by this case where the tumor recurred 1 month after initial resection, reoccurred within 1 week of reoperation, and ultimately led to the patient's death from intracranial hypertension—and has a poor prognosis (median survival often < 12 months) with no standardized treatment guidelines. Given this, immediate postoperative adjuvant chemoradiotherapy (referable to regimens for other WHO grade III meningiomas, such as 50–60 Gy radiotherapy plus temozolomide chemotherapy) is imperative; molecular testing (e.g., detecting BRAFV600E, which was partially positive in this case) and participation in targeted therapy or clinical trials also hold promise for improving outcomes. Ultimately, more large-scale studies and trials are needed to establish evidence-based guidelines and better address this disease's dismal prognosis.
